# Making a Financial Case for the Geriatric Surgery Verification Program

**DOI:** 10.1097/AS9.0000000000000439

**Published:** 2024-05-13

**Authors:** Mark R. Katlic, Joshua Wolf, S. Jasmine Demos, Ronnie A. Rosenthal

**Affiliations:** From the * Center for Geriatric Surgery, Department of Surgery, Sinai Hospital, Baltimore, MD.

## Abstract

Mini abstract

The financial benefits of instituting the American College of Surgeons Geriatric Surgery Verification Program far exceed the costs, with the added benefits of enhanced patient satisfaction and improved staff morale.

## INTRODUCTION

One of the younger standards-and-verification quality programs of the American College of Surgeons is the Geriatric Surgery Verification (GSV) program.^1^ Residing in the same college division as similar programs related to cancer, trauma, breast, and more, the GSV offers hospitals the opportunity to establish 32 evidence-based standards for the care of older adult surgery patients and to be recognized for excellence in that care.

Since its rollout in 2019, ten hospitals have been verified, 1 has been reverified, and 54 have applied and are working toward verification. Although momentum has been building, it has been slow, with this group of committed institutions representing approximately 1% of the hospitals in the United States. These are sobering statistics, particularly for a program intended to be applicable to hospitals of any size and location. Older adults live everywhere, and travel to a referral center for surgery would be unpalatable to most and problematic for many.

One of the greatest barriers to implementing the GSV is purely financial; its practical corollary is the necessary approval by the “C Suite” to go ahead. These chief executives are understandably concerned about their hospitals’ fiscal viability and often favor other competing programs with greater perceived value or low perceived cost. Many hospitals have surgeon champions and nurses eager to get started on the GSV but they have been unable to make a strong enough financial case. There is a sense that the landscape has changed (even greater focus on cost) while the demographic challenge has not (increasing numbers of older adults needing surgery).

This article examines the financial cost *versus* benefits (Table 1) of the GSV Program.

**TABLE 1.** Costs and Benefits of Geriatric Surgery Verification Program

Costs	Benefits
GSV program fee	↓ Length of stay
Program coordinator salary and benefits	↓ Postoperative delirium
Nursing time	Program coordinator revenue
Information technology time	↓ Readmission
	↓ Complications
	Patient satisfaction
	↑ Staff morale
	↓ Loss of independence
	Palliative care discussions
	Marketing

## COSTS OF THE GSV

### GSV Program Fee

The annual fee paid to the American College of Surgeons for the Comprehensive Level 1 Program is $15,000. For rural or government hospitals it is $7500 annually. Hospitals, however, are encouraged to begin at a commitment level, $2500 annually, as this recognizes that it takes time to build the program, and commitment level offers the full resources of the college, including an implementation course, educational materials, and coaching.

### Program Coordinator Salary and Benefits

The GSV does not require a specific educational background or full-time equivalent (FTE) commitment of the coordinator. Programs typically assign the role 0.5 to 1.0 FTE. This individual may be a nurse practitioner (NP), physician assistant, nurse, or administrator. One advantage of an NP is that a majority of states allow an NP to bill for clinical services, such as consults and daily visits (see *Benefits* section below).

The average United States salary and benefits of a 1.0 FTE NP are $153,000. A registered nurse’s 1.0 FTE salary and benefits are $96,760. Many programs have coordinators already performing other roles, thereby assigning less percentage FTE to the GSV and correspondingly less cost.

### Nursing Time

Nurses on any unit that cares for postoperative surgery patients age 75 or older will be adding an additional “vital sign”, a brief screen for postoperative delirium, such as the Confusion Assessment Method, to their routine tasks each day or each shift. This screening evaluation takes about 3 minutes, performed during medication administration or taking vital signs. Nurses will also be required at hire and at each verification cycle to be educated in basic geriatric principles, generally via an hour-long online course.

### Information Technology Time

Leveraging information technology (IT) is the single best strategy for building the GSV program. IT can help with documentation, which is necessary for vulnerability screens, goals of care discussion, multidisciplinary committee discussion, the state of the patient at discharge, and more. Order sets may be constructed for delirium prevention and care, multimodal opioid-sparing pain management, and more. Data should be collected with respect to length of stay (LOS), postoperative delirium rates, readmissions, and mortality.

## BENEFITS OF THE GSV

### Decreased Length of Stay

Several hospitals have reported significant decreases in LOS after the institution of GSV or similar programs. Jones et al^2^ found that 162 patients in the GSV group, compared to 308 patients in a matched comparison group, had a median LOS of 4 days rather than 5 days, *P* < 0.01. At Duke University, 183 patients in their perioperative optimization of senior health program had a median LOS of 4 days compared to 6 days in the 143 patient control group, *P* < 0.001.^3^ A similar program for vascular surgery patients in London reported mean LOS of 3.32 in 85 patients *versus* 5.53 days in 91 control patients, *P* < 0.001.^4^ At Johns Hopkins Bayview Medical Center, the geriatric surgery pathway patients who were frail, 154 patients, experienced a similar decreased LOS, incidence rate ratio 0.97, *P* < 0.001.^5^

Each additional day in the hospital may add variable costs of $650 to $1000. If 100 older surgical patients were spared that additional day, the savings would be $65,000 to 100,000.

### Decreased Postoperative Delirium

Measured rates of postoperative delirium vary widely among hospitals, as much as 8.5 fold,^6^ depending on how it is being diagnosed and how often screening is carried out. Some hospitals have reported higher rates of documented delirium after the institution of a geriatric surgery program despite fewer overall complications and shorter LOS, suggesting that delirium is simply being diagnosed more.^3^ Partridge et al^4^ showed a reduction from 24% to 11%, *P* = 0.018, in their vascular surgery patients after the institution of a geriatric surgery program. Chen et al^7^ reported a 56% reduced odds of delirium after abdominal surgery in patients managed with a protocol similar to the GSV, with an associated 2-day decrease in LOS.

Each episode of postoperative delirium will cost a hospital an average of $20,327 additional per admission.^8^ For every 50 postsurgical patients, the GSV may prevent 5 of the potential 10 cases of delirium, resulting in savings of over $100,000. Delirium will likely prolong LOS, result in functional and cognitive decline, some of which may be permanent, and increase the chance of institutionalization and death.

### Nurse Practitioner Revenue

If a hospital employs an NP as a coordinator, much of his or her salary and benefits may be covered by clinical revenue. The majority of states allow NPs to bill independently, resulting in income from inpatient and outpatient consults and daily visits. As an example, if an NP performed 5 outpatient consults per week at a billed rate of $90 each, the yearly revenue would be $22,500; 5 inpatient consults per week at $210 each would result in $52,500 yearly; 10 inpatient daily visits per week at $40 each would result in $20,000 yearly. Total charges would be $95,000 in a year.

### Decreased Readmission

McDonald et al^3^ reported lower readmission rates in patients included in the geriatric surgery optimization program *versus* controls: 2.8% *versus* 9.9%, *P* = 0.007 at 7 days and 7.8% *versus* 18.3%, *P* = 0.004 at 30 days.

### Decreased Major Complications

Patients in the perioperative optimization of senior health group at Duke experienced fewer complications such as cardiogenic or hypovolemic shock, bleeding, and ileus, 44.8% *versus* 58.7%, *P* = 0.01.^3^ The geriatric surgery program in London vascular surgery patients resulted in significantly fewer cardiac complications and bladder/bowel complications.^4^ The odds ratio for major complications among geriatric surgery pathway patients at Johns Hopkins Bayview was 0.63, a 19% reduction, *P* < 0.001.^5^

When complications occur, they result in additional hospital costs ranging from $10,000 to $50,000 and as high as $300,000 depending on the nature of the complication.^9^

### Improved Patient Satisfaction and Staff Morale

Public opinion about surgery in older adults includes distrust of surgery and complaints of ineffective communication and allowing unrealistic expectations; recommended solutions are multidisciplinary teams and patient-centered communication.^10^ All of these themes are ameliorated by the GSV. In our experience, every older adult patient has welcomed our “leave no stone unturned” evaluation and increased postoperative attention.

No quality improvement program that we have built has generated the devotion among nursing and administrative staff that the GSV has, for although we were caring well for our older adults we were not caring optimally. Our intensive care team, for example, stated that they have desired a number of these standards for years but needed the push of the GSV program. Just beneath the surface and not always expressed openly, we have been hungry for these changes.

### Miscellaneous Salutary Results

The GSV and similar programs have resulted in decreased loss of independence and increased chance of going home following surgery.^3,5^ Routine goals of care discussions result in more appropriate use of palliative care, resulting in nonoperative management, comfort care, or hospice when indicated. Hospitals may choose to market their investment in the care of older adults, resulting in increased referrals.

The reasons for these benefits are straightforward and intuitive: early recognition of each older adult’s specific vulnerabilities, multidisciplinary care, careful medication management, enhanced communication, and early discharge planning. The GSV provides a well-researched, structured program, “off the shelf”, for providing better care in addition to the extensive resources of the American College of Surgeons to assist implementation.

## CONCLUSIONS

The financial benefits of instituting the American College of Surgeons GSV program far exceed the costs, with the added benefits of enhanced patient satisfaction and improved staff morale.
